# Genome-Guided Identification of Surfactin-Producing *Bacillus halotolerans* AQ11M9 with Anti-*Candida auris* Potential

**DOI:** 10.3390/ijms251910408

**Published:** 2024-09-27

**Authors:** J. Francis Borgio, Rahaf Alhujaily, Aqeelah Salman Alfaraj, Maryam Jawad Alabdullah, Rawan Khalid Alaqeel, Ayidah Kaabi, Rahaf Alquwaie, Norah F. Alhur, Reem AlJindan, Sarah Almofty, Dana Almohazey, Anandakumar Natarajan, Tharmathass Stalin Dhas, Sayed AbdulAzeez, Noor B. Almandil

**Affiliations:** 1 Department of Genetic Research, Institute for Research and Medical Consultations (IRMC), Imam Abdulrahman Bin Faisal University, Dammam 31441, Saudi Arabia; fbalexander@iau.edu.sa (J.F.B.);; 2 Department of Epidemic Diseases Research, Institute for Research and Medical Consultations (IRMC), Imam Abdulrahman Bin Faisal University, Dammam 31441, Saudi Arabia; 3 Summer Research Program, Institute for Research and Medical Consultations (IRMC), Imam Abdulrahman Bin Faisal University, Dammam 31441, Saudi Arabia; rahafalhujaily@gmail.com (R.A.);; 4 Master Program of Biotechnology, Institute for Research and Medical Consultations (IRMC), Imam Abdulrahman Bin Faisal University, Dammam 31441, Saudi Arabia; 2230500195@iau.edu.sa; 5 Department of Microbiology, College of Medicine, Imam Abdulrahman Bin Faisal University, Dammam 40017, Saudi Arabia; raljindan@iau.edu.sa; 6 Department of Stem Cell Research, Institute for Research and Medical Consultations (IRMC), Imam Abdulrahman Bin Faisal University, Dammam 31441, Saudi Arabia; saalmofty@iau.edu.sa (S.A.); daaalmohazey@iau.edu.sa (D.A.); 7 Department of Education, The Gandhigram Rural Institute (Deemed to be University), Dindigul 624302, India; 8 Centre for Ocean Research (DST-FIST Sponsored Centre), MoES-Earth Science & Technology Cell, Sathyabama Institute of Science and Technology, Chennai 600119, India; stalindhas.cor@sathyabama.ac.in; 9 Department of Clinical Pharmacy Research, Institute for Research and Medical Consultations (IRMC), Imam Abdulrahman Bin Faisal University, Dammam 31441, Saudi Arabia

**Keywords:** *Candida auris*, surfactin, antifungal activity, genome sequencing, BGC, *16S rRNA* gene sequencing

## Abstract

The emergence of multidrug-resistant fungi *Candida auris* is a worldwide health crisis connected with high rates of mortality. There is a critical need to find novel and unique antifungal compounds for treating infections of multidrug-resistant fungi such as *C. auris*. This study aimed to illustrate that biosynthetic gene clusters in native bacterial isolates are able to produce antifungal compounds against the multidrug-resistant fungus *C. auris.* It was successfully achieved using large-scale antifungal activity screening, cytotoxicity analysis, and whole genome sequencing integrated with genome mining-guided analysis and liquid chromatography–mass spectrometry (LC/MS). A list of possible gene candidates was initially identified with genome mining methods to predict secondary metabolite gene clusters of antifungal-compound-producing bacteria. Then, gene clusters present in the antifungal-compound-producing bacteria were identified and aligned with the reference genome using comparative genomic approaches. *Bacillus halotolerans* AQ11M9 was identified through large-scale antifungal activity screening as a natural compound-producer against multidrug-resistant *C. auris*, while it was nontoxic to normal human skin fibroblast cells (confirmed using a cell viability assay). The genome (4,197,347 bp) of *B. halotolerans* AQ11M9 with 2931 predicted genes was first mined for detecting and characterizing biosynthetic gene clusters, which revealed 10 candidate regions with antifungal activity. Clusters of AQ11M9 encoded non-ribosomal peptide synthase (NRPS) (bacilysin, bacillibactin, paenibactin, surfactin, plipastin, and fengycin) and polyketide (macrobrevin). The presence of gene clusters with anti-*C. auris* activity, and surfactin identified through LC/MS, from AQ11M9 suggests the potential of utilizing it as a source for a novel and powerful anti-*C. auris* compound.

## 1. Introduction

*Candida auris* is an invasive multidrug-resistant (MDR) fungus which was first reported in 2009 in Japan and has since been recognized globally as a serious threat that is difficult to manage and treat [1,2,3,4]. Five clades of *C. auris* isolates were reported from various countries, with an increasing prevalence specifically in immunocompromised patients or those experiencing prolonged hospitalization [1,3,5,6,7]. It exhibits extensive resistance to various antifungal drugs such as azoles, echinocandins, and polyenes [3]. Furthermore, it is challenging to detect *C. auris* using conventional mycological techniques [8]. Drug repurposing strategies and investigations involving nanomaterials have been proposed as alternative options to traditional antifungal medications for inhibiting the growth of *C. auris*, but promising drugs are still needed for appropriate management of *C. auris* [9,10,11]. 

The emergence of multidrug-resistant *C. auris* makes it a candidate for drug discovery and novel development of targeted drugs for treatment. Many inhibitory compounds have been identified by in vitro studies. Ibrexafungerp, a glucan synthase inhibitor, exhibited anti-*C. auris* activity, with a minimum inhibitory concentration (MIC) of 50% inhibition at 1 mg/L [12]. Currently, the efficacy and safety of ibrexafungerp are being assessed in a clinical trial (phase 3) to treat candidiasis caused by *C. auris*. Fungerp analog has a wider fungicidal activity among *C. auris* strains, in addition to its potency in low-level pH, which makes it a potential therapy for *C. auris*-associated urinary tract infection [13]. Another investigational arylamidine compound, T-2307, affects fungal mitochondrial membrane integrity [14]. Arylamidine T-2307 has been screened against *C. auris* and showed excellent activity, and its in vivo activity improved survival in mice by 70% after 21 days of infection with reduced fungal burden in kidney tissues compared to the untreated control group [15]. Another anti-*Candida auris* compound is Fosmanogepix (APX001), a prodrug with an MIC that is eight times lower than anidulafungin against 16 isolates. In addition, APX001 produced improved survival and decreased kidney, lung, and brain fungal burdens in neutropenic mice [16]. A tetrazole compound, VT-1598, is a selective inhibitor of Cyp51 enzyme, thus inhibiting ergosterol production. VT-1598 showed prominent in vitro antifungal activity against 100 fungal isolates, with an MIC ranging between 0.03 and 8 mg/L. Treatment with VT-1598 in neutropenic mice resulted in concentration-dependent improvement in survival and lowered kidney and brain fungal burden compared to the control [17]. Another drug in the azole class is ravuconazole, an extended-spectrum triazole found to have a lower MIC compared to fluconazole and amphotericin B [18]. Some plant derivatives (caffeic acid phenethyl ester polyphenols) have also exhibited anti-*Candida auris* activity [19]. Essential oils of *Cinnamomum zeylanicum* leaf and bark inhibited hemolysin factor production and hyphae formations [20]. Essential oils of *Lavandula angustifolia* produced reactive oxygen species and affected genes related to biofilm production in *C. auris* [21]. A natural product from the marine environment, turbinmicin produced from *Micromonospora* sp., exhibited novel targeting of the transfer protein by affecting the vesicle trafficking pathway [22,23]. Nanoparticles were used to reduce the toxicity of Miltefosine and preserve its activity against *C. auris* [24]. Silver nanoparticles were loaded in bacterial cellulose for hydrogel dressing, thus preventing the invasiveness of *C. auris* that occurs in wounds [25].

Opportunistic infection by *C. auris* can be acquired through dry and moist environmental surfaces in healthcare settings, according to hospital surveillance studies [7,26]. The synthesis of several virulence factors, such as hydrolytic enzymes and the capacity to form biofilms, which support *C. auris’*s prolonged colonization on environmental surfaces and human skin, is also related to its long-term survival on hospital surfaces [26]. Therefore, it is imperative to improve hospital infection control methods for managing *C. auris*-related opportunistic infection by using efficient disinfectants and antiseptics [27]. Hence, there is a critical need to find novel and unique antifungal compounds for treating *C. auris* infections. Native microorganisms are favorable resources for discovering anti-*C. auris* products. Whole-genome sequencing analysis of *Limosilactobacillus fermentum* primarily revealed activity against *Candida* species [28]. *Lactobacillus crispatus* and *Lactobacillus gasseri* isolates exhibited significant reduction in *C. albicans* adhesion [29]. Anti-*Candida albicans* metabolites were observed from *Bacillus velezensis* JT3-1 through secondary metabolite cluster screening with genome analysis [30]. Genome mining showed that *Streptomyces* sp. strain S-2 with anti-*Fusarium* action encompassed 39 biosynthetic gene clusters (BGCs) [31]. Several studies have revealed the significance of genome analysis towards identifying biosynthetic potential among bacterial isolates [31,32,33,34]. However, genome studies of bacteria with anti-*C. auris* activity are scarce. The objective of this work was to conduct a comprehensive screening of antifungal activity on a broad scale using bacterial isolates of indigenous origin. Additionally, the study aimed to elucidate the BGC using whole genome sequencing of bacterial isolates exhibiting anti-*C. auris* properties. This approach involves the integration of genome mining and comparative genomics techniques. Bacterial compounds that exhibit action against multidrug-resistant *C. auris* might potentially serve as a viable choice as a biofactory for natural antifungal compounds.

## 2. Results

### 2.1. Bacterial Strains with Anti-C. auris Activity

**Figure 1:**
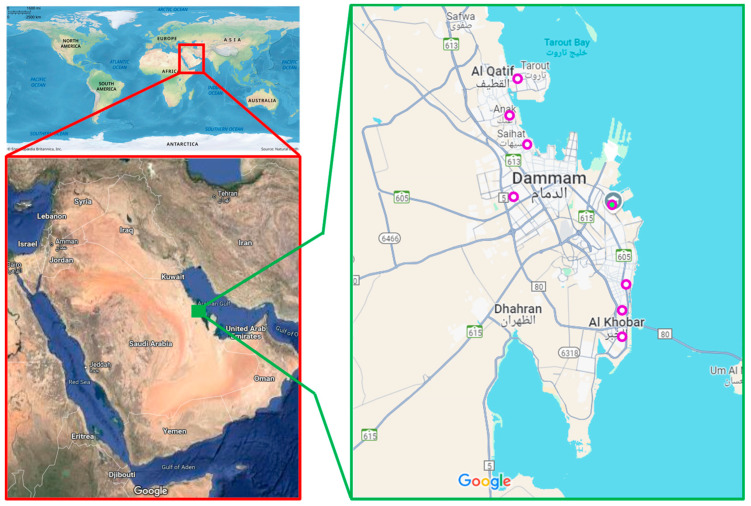
The map displays the geographic location (circled in pink) of the collected samples, including the institute garden (circled in pink-filled green) soil where AQ11M9 was isolated [Courtesy: Encyclopædia Britannica, Inc. (Edinburgh, UK) https://www.britannica.com/science/world-map; Google Maps https://www.google.com/maps (accessed on 17 September 2024).

**Figure 2:**
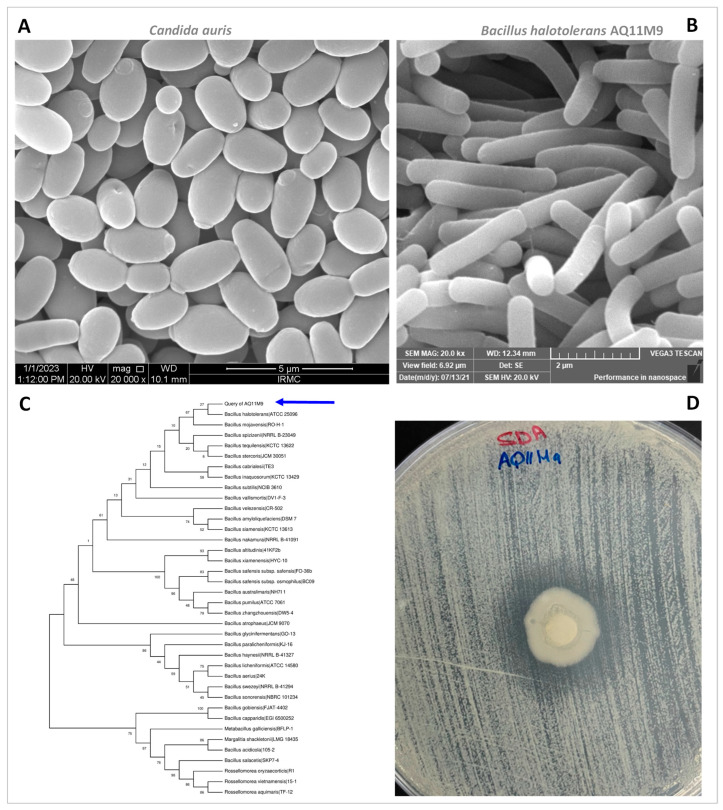
(**A**) Electron-microscopic structure of *Candida auris* (3.083 ± 0.225 × 1.995 ± 0.132 µm) used for the study. Scale bar: 5 μm. (**B**) Electron-microscopic structure of *B. halotolerans* AQ11M9 (2.629 ± 0.151 × 0.675 ± 0.032 µm). Scale bar: 2 μm. (**C**) Phylogenetic tree of *B. halotolerans* AQ11M9. Blue arrow indicates the *16S rRNA* gene sequence of the query AQ11M9. (**D**) Anti-*Candida auris* activity of *B. halotolerans* AQ11M9 with zone of inhibition, 21 to 23 mm. SDA: Sabouraud dextrose agar.

AQ11M9 was isolated from the study institute garden (GPS Coordinates 26°22′51.5″ N 50°11′28.5″ E) soil (Figure 1). The colony characteristics were observed after isolating AQ11M9 from a sample on TSA. The colony appeared as flat, opaque, dry, off-white colonies. AQ11M9 isolate demonstrated the potency of anti-*Candida auris* through an anti-fungal activity test which showed 21 to 23 mm diameter of a zone of inhibition after incubation on SDA for 48 h (Figure 2).

### 2.2. Structural Examination of AQ11M9 and C. auris Utilizing SEM

Representative SEM images of the AQ11M9 strain and *C. auris* are displayed in Figure 2. SEM analysis of *C. auris* of the clinical isolate CA1 appeared as ovoid, spherical cells of 3.083 ± 0.225 × 1.995 ± 0.132 µm in diameter (Figure 2). Characterization of the morphological structure of AQ11M9 using SEM revealed that the bacterial isolate AQ11M9 was rod-shaped and 2.629 ± 0.151 × 0.675 ± 0.032 µm in size (Figure 2); this result confirms that the AQ11M9 strain belongs to bacilli.

### 2.3. 16S rRNA Sequencing of AQ11M9

The AQ11M9 isolate was identified using the *16S rRNA* gene, sequenced through Sanger sequencing. The sequence alignment of the *16S rRNA* gene on the EZBioCloud showed that the AQ11M9 strain (GenBank accession No: MZ619061.1) was similar to *Bacillus halotolerans*, with 100% similarity. A phylogenetic tree constructed with 100 bootstraps showed the evolution history (Figure 2).

### 2.4. In Vitro Cytotoxicity 

**Figure 3:**
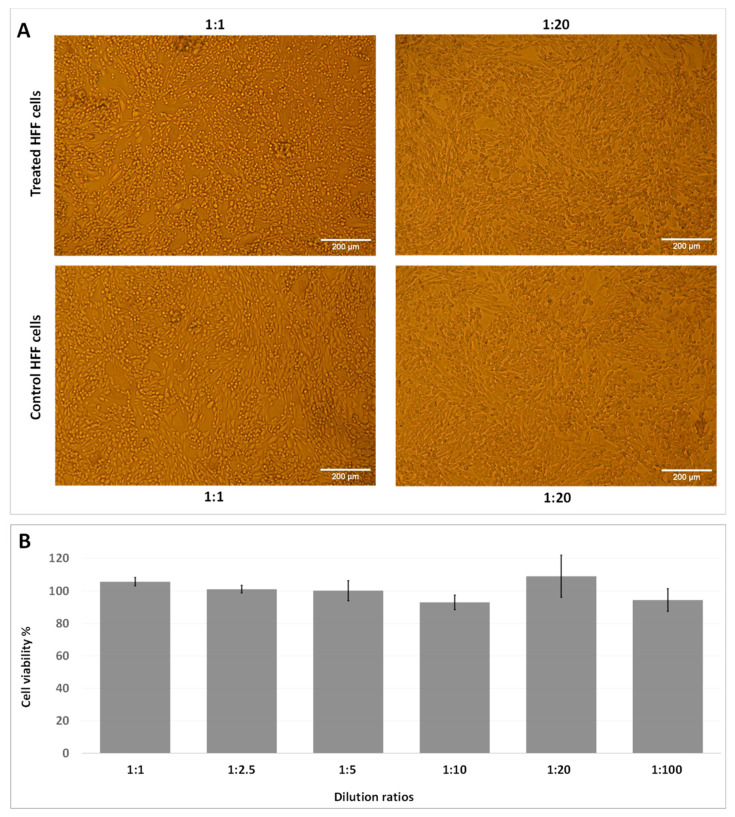
Cell viability of 24 h old broth devoid of *B. halotolerans* AQ11M9 cells against HFF cells: MTT assay was carried out using different dilutions of AQ11M9 broth (1:1, 1:2.5, 1:5, 1:10, 1:20, and 1:100). (**A**) HFF cells (100×) after exposure compared to untreated control. (**B**) Histograms represent the percentage of HFF cell viability, with respect to control cells (100%). No statistically significant (F statistic = 2.4682; *p*-value = 0.0927) differences were observed between exposure and control using one-way analysis of variance followed by post hoc Tukey HSD test.

The percentage cell viability of HFF cells treated with 24 h old AQ11M9 broth (filtered and devoid of bacteria) was determined using an MTT assay with various dilutions. Morphological examination under a light microscope revealed a similar number of cells in the control and in broth-treated cultures at the highest and lowest dilutions (Figure 3A). Moreover, a cell viability assay showed no significant difference between the control and broth-treated cells using one-way analysis of variance followed by a post hoc Tukey HSD test (F statistic = 2.4682; *p*-value = 0.0927) (Figure 3B). These observations clearly demonstrated that the *B. halotolerans* AQ11M9 broth with anti-*Candida auris* properties had no cytotoxic effect on HFF-1 cells.

### 2.5. Identification of AQ11M9 by Whole Genome Sequence

**Figure 4:**
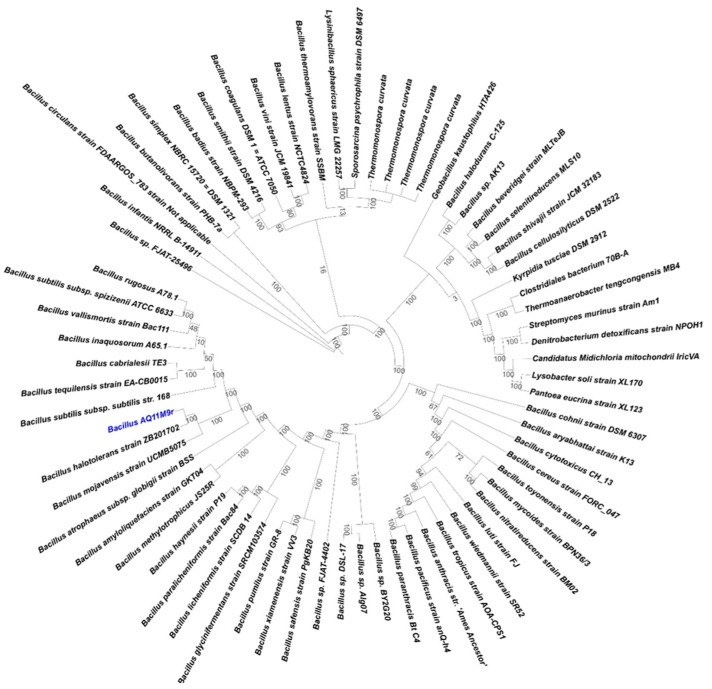
Confirmation of AQ11M9 based on the whole-genome phylogenetic tree of the AQ11M9 genome (coloured blue) using 65 reference genomes at PATRIC with the Mafft alignment program and the RAxML fast bootstrapping branch support method. Single-copy genes are found in the 65 reference genomes, and AQ11M9 is 51. A total of 18625 amino acids and 55875 nucleotides were aligned to construct the whole-genome phylogenetic tree. The list of gene family statistics is presented in Table S1.

Phylogenetic analysis of AQ11M9 based on the sequence of *16S rRNA* gene extracted from the AQ11M9 genome specified that the AQ11M9 strain formed a lineage within the species *B. halotolerans* and *B. mojavensis* and clustered as members of the genus *Bacillus*. To ensure the most similar species, a phylogenetic tree of the AQ11M9 genome was created using PATRIC comparing *B. halotolerans* and *Bacillus mojavensis* through a 100-gene alignment request; however, 51 single-copy genes were found in the AQ11M9 genome and 65 reference genomes (Figure 4). This revealed that the AQ11M9 genome is closely related to *B. halotolerans*. To further determine the taxonomic affiliation of AQ11M9, the whole genome of AQ11M9 was uploaded to TYGS for AQ11M9-genome-based taxonomic classification, which assigned *B. halotolerans* as the expected organism of the AQ11M9 strain. However, when the sequence was analyzed using PATRIC and EPI2ME, it was identified as being very similar to the unclassified PAMC26543 strain followed by the *B. halotolerans* V84-19 strain (Figure S1), suggesting that AQ11M9 could be a new strain of *B. halotolerans* based on these different results. For more confirmation, the comparison between these three organisms was carried out based on genome regions and genome proteome.

### 2.6. Genome Analysis and Comparative Study

The genome assembly of the Oxford-Nanopore-based long-read sequence unearthed the AQ11M9 (reads analyzed: 10,079; total yield: 109.7 Mbases; average quality score: 10.70; average sequence length: 10,881) genome with 4,197,347 bp and an average guanine + cytosine (G + C) content of 43.73%. Furthermore, the AQ11M9 genome was assembled into one contig with N50s of 4,197,347. The annotation predicted 5623 coding sequences (CDS) and 2931 predicted genes (unique); the remaining characteristics are delineated in Table 1.

**Figure 5:**
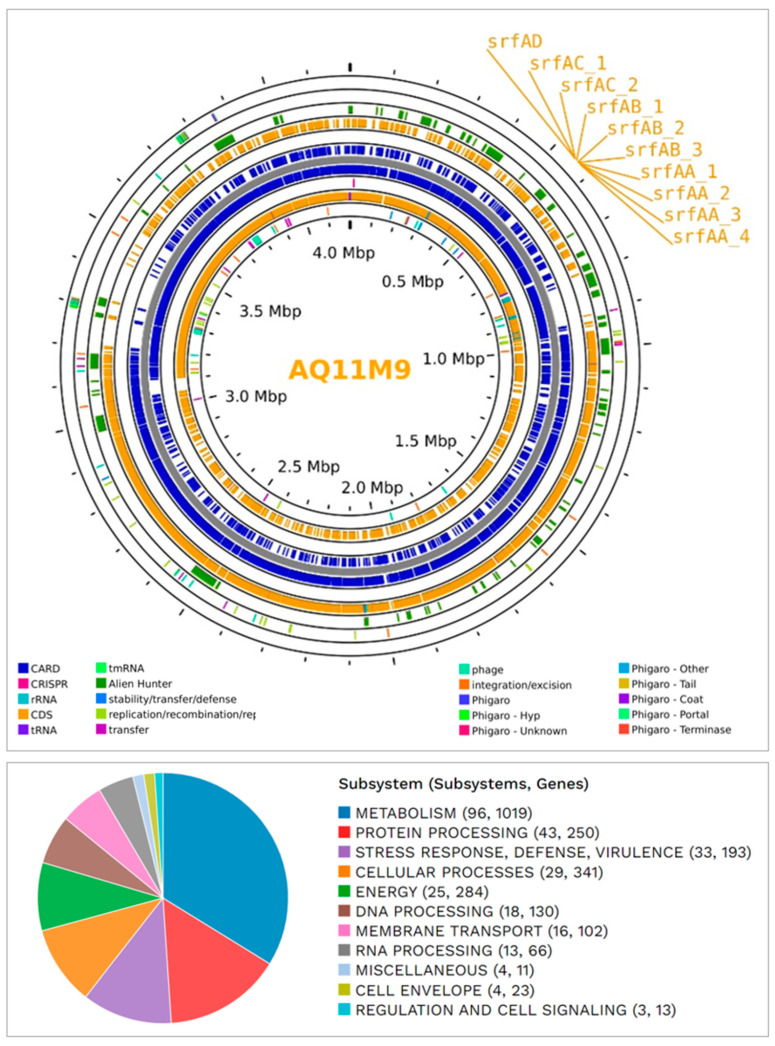
(**Top**) Circular genome map for genome information of *B. halotolerans* AQ11M9. The gene clusters associated with surfactin biosynthesis were identified in the genome of strain AQ11M9 are indicated. (**Bottom**) Functional annotation of the AQ11M9 genome.

**Figure 6:**
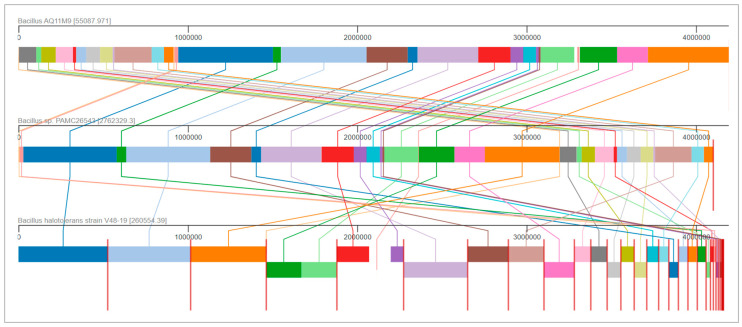
Comparative analysis of AQ11M9 genome. Genome comparisons represent homologous regions (colored similarly) between AQ11M9, *B. halotolerans* strain V48-19, and PAMC26543. Each block in the MAUVE analysis depicts a part of a genome’s sequence that corresponds to a region of another genome, suggesting that the region is homologous and internally devoid of genomic rearrangements. Homologous locally collinear blocks between genomes AQ11M9, *B. halotolerans* strain V48-19, and PAMC26543 are identified by a similar color and linked together through the use of a connecting line. Blocks above the central line in *B. halotolerans* strain V48-19 indicate forward orientation relative to *B. halotolerans* AQ11M9 genome; blocks below the central line in *B. halotolerans* strain V48-19 indicate reverse-complement orientation on the regions that align with the *B. halotolerans* AQ11M9 genome.

**Figure 7:**
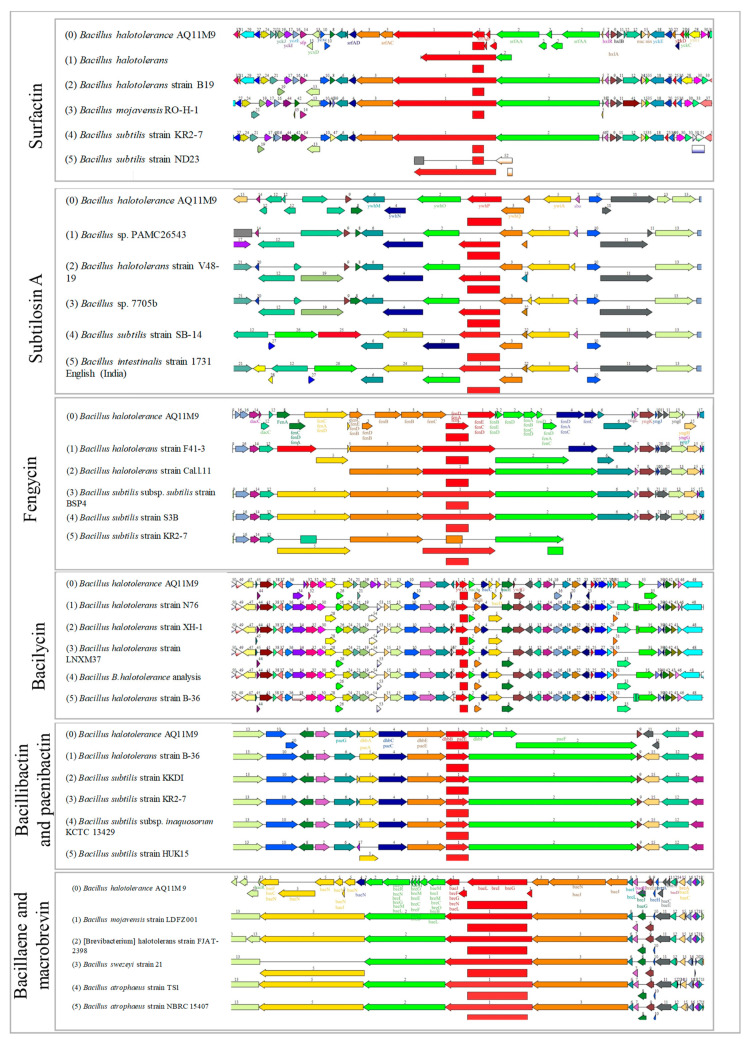
Genetic organization of the identified putative BGCs in genome of *B. halotolerans* AQ11M9 using PATRIC. Conservation of six clusters in the genome of *B. halotolerans* AQ11M9 with other species in the same genus. The orientation of the gene in BGCs is indicated by the direction of the arrow, representing both the forward and reverse orientations.

The contiguous sequence of the AQ11M9 genome was utilized for circular genome mapping. This mapping was performed to illustrate the subsystem, function, and overall characteristics of the AQ11M9 sequence (Figure 5 and Figure S2). The genomic similarities between the AQ11M9 strain and two species, namely the unclassified PAMC26543 and the *B. halotolerans* V84-19 strain, prompted us to conduct a comparative analysis of their genomes. Figure 5 displays the MAUVE comparison of three entities, namely AQ11M9, PAMC26543, and the *B. halotolerans* V84-19 strain. Moreover, the genome comparison showed that synteny blocks among the AQ11M9, PAMC26543, and *B. halotolerans* V84-19 strain are longer, with regions containing conserved household genes and showing high nucleotide conservation. However, the beginning of the chromosomal regions revealed variable genes with biosynthetic clusters (Figure 6 and Figure S2). The synteny blocks and homologous locally collinear blocks between genomes AQ11M9, *B. halotolerans* strain V48-19, and PAMC26543 indicate that AQ11M9 is neither completely similar to PAMC26543 nor to the *B. halotolerans* V84-19 strain, while the proteome comparison showed similarity but not 100% similarity (Figure S3). Closer review revealed that chromosomal regions with variable genes (Figure 7) contain surfactin gene clusters (Figure S2A), fengycin, plipastatin, and mycosubtilin gene clusters (Figure S2E).

### 2.7. Secondary Metabolite and Biosynthetic Gene Detection

The whole genome of *B. halotolerans* AQ11M9 was subjected to structure prediction from secondary metabolite BGCs to predict the metabolites in support of the observed anti-*Candida auris* activity. The AntiSMASH server successfully detected a total of 38 potential BGCs in the genome of *B. halotolerans* AQ11M9. These clusters exhibited a wide range of similarities, ranging from 3% to 100%. The genome of *B. halotolerans* AQ11M9 has 38 gene clusters that are hypothesized to play a role in the manufacture of secondary metabolites with activity against *C. auris*. These clusters include genes encoding non-ribosomal peptide synthase (NRPS) and enzymes involved in polyketide biosynthesis (PK). Only 10 of the 38 BGC in the *B. halotolerans* AQ11M9 genome had a similarity > 85% with the reference genome (Table 2). Interestingly, these 10 clusters have been reported to have antifungal activity, suggesting that AQ11M9 could produce a variety of natural antifungal compounds associated with its activity against *Candida auris*. The predicted gene cluster in the *B. halotolerans* AQ11M9 genome showed a significant conservation with related strains of the genus *Bacillus* (Figure 7). Structures of bacilycin, bacilaene, bacillibactin, plipastatin and fengycin, mycosubtilin, macrobrevin, surfactin, and subtilosin A were predicted to be the natural antifungal compounds derived from the genome of *B. halotolerans* AQ11M9 using antiSMASH and MIBIG.

Six clusters were predicted to be involved in the *B. halotolerans* AQ11M9 for coding NRPS (bacilysin, bacillibactin, paenibactin, surfactin, plipastin, and fengycin), and the other four were related to the biosynthesis of PK (macrobrevin): both NRPs and PK (mycosubtilin and bacillaene) and sactipeptide (subtiloson A) (Table 3). BGC comparison between AQ11M9 and closely related strains (*B. halotolerans* strain V48-19 and PAMC26543) showed that genes associated with NRPs and PK biosynthesis were characteristically predicted on the genomes of both AQ11M9 and PAMC26543 strains; the genes for the predicted NRPs and PK products found in both AQ11M9 and PAMC26543 were not found in V48-19 (Table 3). The aforementioned results, accompanied by comprehensive taxonomical investigations, provide evidence for the necessity of future reassignment of the AQ11M9 strain. 

Based on antiSMASH and MIBIG, bacilysin-, fengycin-, and plipastatin-associated clusters showed 100% sequence uniqueness with *Bacillus velezensis* FZB42, including seven (*bacA*, *bacB*, *bacC*, *bacD*, *bacE*, and *bacF*), five (*fenABCDE*), nine (*yngA*, *B*, *C*, *D*, *E*, *F*, *G*, *H*, and *yngI*), and five (*ppsA*, *ppsB*, *ppsC*, *ppsD*, and *ppsE*) subunit genes, respectively, while the surfactin gene (*srfAA*, *srfAB*, *srfAC*, and *srfAD*) cluster exhibited 86% sequence identity with *Bacillus velezensis* FZB42. However, the genome of AQ11M9 was shown to lack (*aat* and *XY022*) genes responsible for amino transferase and XY022 protein synthesis. The bacillaene showed 92% similarity with *B. velezensis* FZB42, which included *baeJ*, *baeL*, *baeM*, *baeN*, and *baeR* genes. On the other hand, the subtilson A-associated genes exhibited 87% identity with *B. spizizenii* ATCC 6633, which includes the sob gene. The baenlibactin genes exhibited 100% identity with *Paenibacillus elgii* B69, which includes the *baef* gene. The bacillibactin had 100% identity with *B. subtilis* subsp. *subtilis* str. 168 with five (*dhbA*, *dhbC*, *dhbE*, *dhbB*, and *dhbF*) genes. The macrobrevin was associated 100% with *Brevibacillus* sp. Leaf182, which contains *breC*, *breG*, *breM*, *breE*, *breA*, *breN*, *breB*, *breO*, *breD*, and *breF* genes. All gene names of each cluster are listed in the genome map (Figure S2).

The AQ11M9 genome sequence was subjected to NAPDOS analysis, and 27 C-domains (condensation) were found from NRPS clusters (Figure S4). The condensation domains were categorized into classes, and the LCL domain was found in the majority of the sequences. The LCL domain catalyzes the peptide bond establishment, particularly between the L-amino acids. The screening and analysis for type I PKS were carried out in nine KS domain regions. Most of the domain regions were categorized as modular type I PKS in the *B. halotolerans* AQ11M9 genome. The results of NAPDOS revealed four domain classes: DCL, Starter, LCL, and epim (Table 4).

### 2.8. LC/MS Analysis of AQ11M9’s Secondary Metabolites

**Figure 8:**
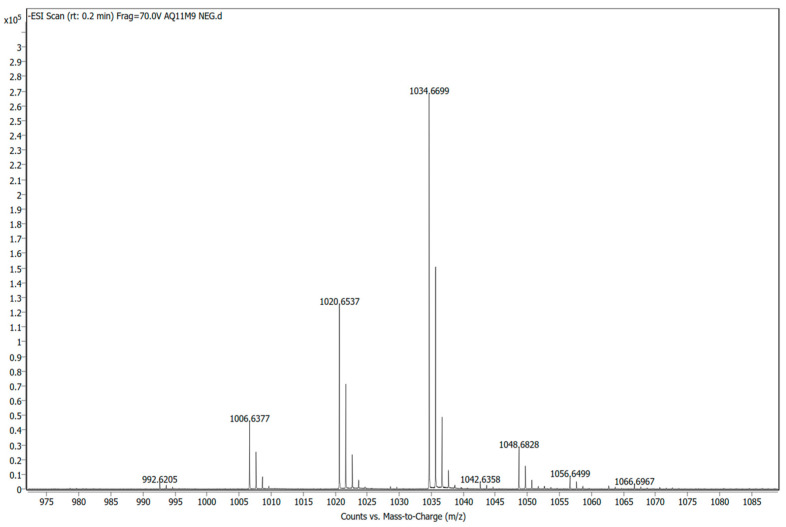
LCMS analysis of secondary metabolite produced by *B. halotolerans* AQ11M9.

Preliminary validation of the biosynthetic gene-associated products detected using genome mining was carried out on the cell-free broth of AQ11M9 using LC/MS. The secondary metabolite produced by *B. halotolerans* AQ11M9 analyzed using LC/MS revealed the presence of surfactin-like molecules with a mass-to-charge ratio = 1034.6699 *m*/*z* (Figure 8) and a corresponding molecular weight = 1036.3 g/mol.

## 3. Discussion

Since its discovery in 2009, the multidrug-resistant fungus *C. auris* has garnered significant attention as a noteworthy pathogen due to its challenging diagnostic and therapeutic characteristics [1,2,3,4,8,35]. *C. auris* CA1 was reported to have many drug-resistant mutations such as the silent variants G534G, Y584Y, and F585F; the missense variants H608Y, P611S, and A640V in the *TAC1B* gene; and the F132Y, K143R, and K152K mutations in the *ERG11* gene [9]. Various approaches have been used in the search to identify possibilities to develop novel treatment strategies for treating infection caused by various clades of *C. auris* among immunocompromised patients and those who have been hospitalized for a long time [1,3,5,6,7,9,10,36,37]. The anti-*Candida auris* potential of nanoemulsions is still undergoing toxicity studies to ensure their effectiveness [38,39]. Therefore, the need for novel potential compounds persists for managing *C. auris*-related opportunistic infection and spread [27]. Bacterial strains derived from diverse origins exhibit considerable potential as valuable resources for the exploration of anti-*Candida* properties [29,30,40,41,42,43,44]. Anticandidal activities of *Lactobacillus* observed against *Candida albicans* suggest that the metabolites of *Lactobacillus* can be a promising therapeutic alternative for treating candidiasis by *C. albicans* [45,46,47,48]. Nevertheless, there is a lack of comprehensive research about the metabolites produced by *Lactobacillus* and their impact on *C. auris* [48].

The primary objective of this study was to identify and evaluate indigenous bacterial isolates that produce metabolites with anti-*C. auris* properties. Through our efforts, we were able to isolate a novel strain of bacteria, presumably *B. halotolerans* AQ11M9. The study directly used trypticase soy agar for isolating bacteria; for a broader range of discovery of bacteria, different culture media shall be considered. Many reports have emphasized the use of genome mining in discovering bacterial isolates with anti-*Candida albicans* metabolic potential; however, there are no reports to date for *C. auris* [28,30,31,32,33,34]. This study found that *B. halotolerans* AQ11M9 was associated with 10 BGCs that might be associated with producing antifungal natural compounds with anti-*Candida auris* activity. These findings were in line with the *antiCandida* properties of the compounds produced *by Bacillus velezensis* JT3-1 [30] and *Limosilactobacillus fermentum* [28]. A newly characterized species, *B. halotolerans* [49], was reported to possess antimicrobial activity against fungal pathogens, mostly against plant pathogens [50,51,52,53,54,55]. There is a lack of documented findings in the existing literature regarding the potential of *B. halotolerans* as an anti-*Candida auris* agent. This study describes the anti-*Candida auris* activity of the novel *B. halotolerans* AQ11M9 strain from a soil in the Dammam region of Saudi Arabia. The genome of AQ11M9 was sequenced; it contains 4,197,347 bp with 43.73% G + C content. The anti-*Candida auris* activity of AQ11M9 would be fascinating to investigate in future drug candidate research if the novel bacterial strain may be used to produce drugs that are effective against MDR fungus. Moreover, AQ11M9 was classified as *B. halotolerans* AQ11M9, as the 16S rDNA gene sequences were similar to *B. halotolerans* ATCC 25096 (GenBank: LPVF01000003.1) [49]. To achieve accurate identification of the isolate, we conducted whole genome sequencing utilizing long-read nanopore sequencing. This portable equipment generates reads with a maximum length and has the capability to display sequences in real time [56,57,58,59]. Long-read sequencing was preferred, as the other short-read sequence techniques are suboptimal due to the low integrity of most of the assembled region from short-reads and because they provide insufficient phylogenetic resolution [60]. Our study generated a single contig for the AQ11M9 genome, as the long-read nanopore sequencing was successful, with maximum reads in N50. Oxford Nanopore Technologies’ nanopore long-read sequencing is one of the most widely used sequencing technologies for microbial genomics to generate ~100 kb for ultra-long reads [61,62]. The number of predicted BGCs with 100% similarity might vary depending on the sequencing technique and single contig that results [31]. We ensured high coverage together with long-read nanopore sequencing in the current work. Through the utilization of this approach, we successfully sequenced genomes of superior quality, characterized by the absence of extensive contig-spanning BGCs, and as we obtained a single contig, this also increased the reliability of antiSMASH’s accurate prediction [63].

The *Bacillus halotolerans* MS50-18A strain, which is active against fungal phytopathogens, was isolated from saline soil; this indicates that soil is one of the reservoirs for *Bacillus halotolerans* [50]. The draft genome of *B. halotolerans* MS50-18A contains 4215 genes in a 4.06 Mb sequence; however, *Bacillus halotolerans* AQ11M9 in our study revealed a genome size of 4.19 Mb and 5623 genes [50]. Another study reported 4,141,192 bp for *B. halotolerans* Hil4, which shows bioactivity against the pathogenic fungus *Botrytis cinerea* [52]. The *B. halotolerans* Cal.l.30 (active against *Botrytis cinerea*) genome was reported to have nine scaffolds and a size of 4.20 Mb [53]. The *B. halotolerans* Cal.l.30 genome is 8711 bp larger than our *B. halotolerans* AQ11M9 genome [53].

Our study detected bacilysin, similarly to an earlier study on the *B. halotolerans* MS50-18A strain, with antifungal activity on various phytopathogens, though our study focused on the human pathogen *C. auris* [50]. The *B. halotolerans* Cal.l.30 genome was reported to contain lipopeptides, mojavensin A and surfactin, fengycin, L-dihydroanticapsin, bacillibactin, and bacillaene isoforms in correlation with our study for the presence of most these metabolites in the AQ11M9 genome [53]. *B. halotolerans* Cal.l.30 is used as an antifungal agent in plants. These studies are aligned with our cytotoxicity studies in confirming that *B. halotolerans* AQ11M9 could be a worthy candidate for producing drugs against MDR *C. auris*. [53]. Expanding the analysis of hemolytic activity with additional eukaryotic cell line assays at varying dilution factors shall enhance evaluation of cytotoxicity. One of the most significant findings in our study was the presence of fengycin and surfactin clusters in AQ11M9, which are members of the cyclic lipopeptides (CLP) family that globally play important roles against pathogens. Plipastatin and fengycin are related molecules with a difference on the peptide moiety at the D-tyrosine position [64,65]. Heterologous expression of the corresponding gene identified in the study is needed to confirm the production of the listed compounds. LC/MS analysis of secondary metabolites produced by *B. halotolerans* AQ11M9 identified the production of surfactin. This observation is in line with the earlier studies on the antifungal activities of bacterial surfactin [66]. Furthermore, elaborative confirmative studies are needed to ensure the presence of all the other listed compounds using various advanced analyses. 

## 4. Materials and Methods

### 4.1. Isolation of Bacteria

Samples were collected from a wide range of sources to search for bacterial strains exhibiting antimicrobial action against *C. auris*. All the samples were collected in sterile containers. Prior to isolation of bacteria, each sample was processed, depending on the nature of the sample, to enrich bacteria. The fine particles, which included soil samples, underwent a series of dilutions and were subjected to the spread-plate technique using trypticase soy agar (TSA) (MoleQule-On, Auckland, New Zealand). The samples were then incubated at a temperature of 37 °C for a duration of 24 h. The solid samples underwent fragmentation into smaller fragments, which were subsequently immersed in a solution of trypticase soy broth (TSB). The hard samples underwent a washing procedure utilizing sterile distilled water and were thereafter enriched by employing TSB. On the other hand, the liquid samples were subjected to direct processing for serial dilution using TSB. Larger objects were inspected for surface bacteria using swabbing followed by serial dilution using TSB. All samples were incubated for 24 h at 37 °C. Incubated TSB tubes with turbidity were further processed for isolation of bacteria by the streaking method using TSA, and isolated colonies were obtained. The process of isolation from the turbid broth was performed in triplicate, and the broth was incubated at 37 °C for 24 h to obtain more bacterial isolates. Once the colony features were confirmed, efforts were made to ensure the isolation of well-isolated colonies for the purpose of obtaining pure cultures. All the 24 h old isolates were stored in 50% glycerol mixed with trypticase soy broth and stored at −80 °C.

### 4.2. Anti-Candida auris Activity Screening

The potency of bacteria to be anti-*Candida auris* was determined by the Kirby–Bauer disk diffusion method. The *C. auris* strain CA1 was reconfirmed by sequencing the ITS region and *18S rRNA* gene [9,67]. *C. auris* was incubated at 37 °C for 24 h to achieve the 0.5 McFarland standard. Bacterial isolates were prepared from a single bacterial colony that originated in 24 h old culture. The disk was submerged in broth (TSB) that had been cultured for 24 h, allowing for the absorption of bacterial products. The disk that was fully submerged was placed onto Sabouraud dextrose agar (SDA) (MoleQule-On, Auckland, New Zealand). The SDA was previously inoculated for a duration of 48 h with *C. auris* using the spread technique to ensure complete coverage of the entire plate. Subsequently, the plate was incubated at a temperature of 37 °C for a duration of 24 to 48 h. The antifungal capability of the bacterial isolates against *C. auris* was then assessed in triplicate by measuring the zone of inhibition.

### 4.3. In Vitro Cytotoxic Study

A filter-based extraction procedure was conducted twice on a 24 h TSB culture containing *Bacillus halotolerans* AQ11M9. To eliminate bacterial cells, broth samples were subjected to syringe filtration (Millex-GV filter unit 0.22 µm, Millipore Ltd., Cork, Ireland) twice before using them as a treatment condition. Human foreskin fibroblast cells (HFF-1) were treated with filtered broth samples to assess the cell viability. 

This assessment was carried out by employing the tetrazolium substrate MTT (Sigma-Aldrich (St. Louis, MO, USA), cat. no. M2128) [68]. The HFF-1 cells were cultured in a 96-well plate at a concentration of 1 × 10^4^ cells/mL and were incubated for 24 h prior to exposure to bacterial filtrates. The treatment involved the utilization of filtered broth, whereas a control was established using sterile TSB. Various dilutions of Dulbecco’s Modified Eagle’s Medium (DMEM) and broth were made with the intention of attaining dilution ratios of 1:1, 1:2.5, 1:5, 1:10, 1:20, and 1:100. These dilutions were thereafter incubated with the cells for a duration of 48 h. Then, cells were rinsed with 1× phosphate-buffered saline and the culture medium was substituted with fresh DMEM. Subsequently, 10 µL of MTT reagent was introduced to each well and the cells were maintained at a temperature of 37 °C in a 5% CO_2_ incubator. The measurement of absorbance was conducted using a multimode plate reader (Biotek (Charlotte, VT, USA)) at a wavelength of 570 nm. The statistical significance between the exposure group and the control group was examined through the utilization of one-way analysis of variance (ANOVA) with a subsequent post hoc Tukey HSD test.

### 4.4. Preparation of Bacillus Halotolerans for Scanning Electron Microscopy (SEM)

A square block was obtained from the agar plate of the isolated organism and incubated with 3% glutaraldehyde at 4 °C for 16 h. After the first fixation, the sample was washed with sterile distilled H_2_O three times and then dipped in the second fixative, 1% osmium tetroxide (OsO_4_), for 16 h at 4 °C. Subsequently, the specimen underwent a rinsing process using distilled water, followed by a sequential treatment with acetone solutions of varying concentrations (30%, 50%, 70%, and 100%) for a duration of 10 min. The specimens were placed in pure acetone for the purpose of dehydration. The sample was dried using acetone and carbon dioxide in a Leica EM CPD300 automated critical point drying apparatus. Following the chemical preparation process, the sample was affixed to a metallic stub using double-sided carbon tape and subsequently coated with a layer of gold using the Q150R ES machine. Images of the prepared *B. halotolerans* were captured at different magnifications and analyzed using ImageJ 1.53 t (https://imagej.nih.gov/ij/ (accessed on 20 July 2021) [69].

### 4.5. Preparation of *Candida auris* for SEM

A single colony of *C. auris* was extracted from Sabouraud dextrose agar and introduced into 1 mL of SDB. The mixture was allowed to incubate for a duration of 16 h, after which it was subjected to centrifugation at a speed of 4000 rpm for a period of 10 min. Subsequently, the pellet was gathered and subjected to three washes with phosphate-buffered saline, followed by centrifugation at a speed of 1000 rpm for a duration of 10 min. The fungal sample was treated with 1 mL of a 2.5% glutaraldehyde solution and thereafter stored at a temperature of 4 °C for the duration of one night. On the subsequent day, the fungal sample that had been immersed was subjected to centrifugation at a speed of 1000 rpm for a duration of 10 min. Subsequently, it underwent two rounds of washing using 1× phosphate-buffered saline. Following this, the sample was washed sequentially with ethanol of varying concentrations, namely 30%, 50%, 70%, 90%, and 100%, with each washing step lasting 10 min. The sample was subjected to drying using a Leica EM CPD300 automated critical point drying apparatus, employing a combination of ethanol and carbon dioxide. The specimen was affixed onto a metallic scanning electron microscopy stub and subsequently coated with a layer of gold of approximately 5 nm in thickness. The fungal samples were examined using an SEM (Vega 3, TESCAN, Brno, Czech Republic) with an operating voltage of 20 kV. The digitized photos were subjected to analysis using the software ImageJ [69].

### 4.6. 16S rRNA Gene Sequencing

The first identification of the isolated AQ11M9 strain exhibiting anti-*C. auris* properties was conducted using the *16S rRNA* gene sequencing method as outlined [70]. The purity and concentration of *16S rRNA* PCR amplicon from AQ11M9 were detected via agarose gel electrophoresis, purified and subjected to Sanger sequencing. A phylogenetic tree for the bacterial strain AQ11M9 was then built according to the neighbor-joining method by means of Mega (11.0.7) software and the Ezbiocloud 16S Database [71,72,73]. The neighbor-joining tree for the bacterial strain AQ11M9 was constructed based on bootstrap values with 200 replications. Further taxonomical studies were completed to distinguish the AQ11M9 strain through whole genome sequencing.

### 4.7. Long-Read Whole Genome Sequencing

The whole genomic sequence of the AQ11M9 strain of bacteria was determined utilizing Oxford Nanopore long-read sequencing technology [74], and the coverage and quality of reads were calculated and converted to fastQ files using nanopore minion Epi2ME Desktop Agent 3.7.3. (Oxford Nanopore Technologies-ONT, Oxford, UK) as described previously [70]. Furthermore, using the obtained long-read sequence, the raw data were assembled and annotated using Nanoforms and NanoGalaxy [75,76]. The genome sequences of AQ11M9 were submitted to the type strain genome server (TYGS) and the PATRIC (BV-BRC 3.28.5) for taxonomic categorization based on the complete genome data obtained [77,78].

### 4.8. Genome Analysis

The fastQ data assembled as described previously were processed for genomic analysis and subsequent comparison [78]. The visualization of open reading frames, gene names, and characteristics was accomplished by the utilization of genome mapping techniques. In order to conduct a comparative analysis of genomic rearrangement among the anticipated species from the preliminary analysis, the genomic DNA sequences were aligned utilizing the Mauve technique and subsequently visualized using the PATRIC v3.2.76 software. Whole-genome-based phylogenetic trees using 100 genes were constructed using PATRIC as well [79].

### 4.9. Biosynthetic Gene Cluster (BGC) Detection

The identified anti-*C. auris* activity required an analysis to identify the antifungal compounds, especially anti-*C. auris* metabolites produced by bacterial isolate based on genome sequencing and analysis [70]. Identification of the antifungal compounds produced by this strain was analyzed using the bioinformatics tool antiSMASHs (6.1.1.) [63]. Additionally, comparative gene cluster analysis was completed using MIBiG Version 3.1 [80] based on the GenBank data and was conducted by means of the Cluster Blast and Known Cluster Blast modules. Hidden Markov models (HMMs) were applied to identify a broad spectrum of gene clusters associated with the production of bioactive metabolites in the native strain AQ11M9 genome, which includes encoding polyketides (PKs), non-ribosomal peptides, aminoglycosides, terpenes, and ribosomally synthesized and post-translationally modified peptides (RiPPs). To detect the BGC domains and predicted structure, the NAPDOS 2 bioinformatics portal was used to analyze the genome input of AQ11M9 [81]. The comparison of metabolic regions between AQ11M9 and reference organisms was carried out by using PATRIC software and minimizing the comparative organisms to five organisms [63].

### 4.10. Liquid Chromatography–Mass Spectrometry (LC/MS) Analysis

The secondary metabolite produced by *B. halotolerans* AQ11M9 in the cell-free broth was analyzed using liquid chromatography–mass spectrometry (LC/MS) (quadrupole time-of-flight LC/MS, 6530 LC/Q–TOF, Agilent, Santa Clara, CA, USA) for identifying the biosynthetic products associated with the genes detected using genome mining.

## 5. Conclusions

In conclusion, this study discovered the novel bacterial strain *B. halotolerans* AQ11M9 with potent anti-*Candida auris* activity and suggests that this strain is a strong candidate for further work. The long-read nanopore sequence-based genome, coupled with the feature of microbial genome mining, identified potential BGCs in AQ11M9. The promising secondary metabolism clusters identified in AQ11M9 and surfactin suggested through LC/MS creates the path for future preclinical studies on controlling *C. auris* infection and the structural elucidation of new antifungal compounds and sustainable production. Future studies on the identification of active compounds from AQ11M9, such as surfactin, and AQ11M9’s impact on the development of other pathogenic *Candida* species can expand and lead to pre-clinical trials for its applications in treating candidiasis.

## Figures and Tables

**Table 1:** The list of key characteristics of the genomic sequence pertaining to the AQ11M9 strain.

Features	AQ11M9
Genome size	4,197,347 bp
The quantity of protein coding sequences	5623
The quantity of rRNA genes	30
The quantity of tRNA genes	86
The quantity of tmRNA genes	1
GC content %	43.73
Hypothetical proteins	894

**Table 2:** Overview of BGCs of *B. halotolerans* AQ11M9 strain detected by AntiSMASH.

Region	Predicted Size (bp)	Type of BGC	Most Similar Known Cluster	No. of Genes	MIBIG ID/(Similarity %)
1.1	62,838	NRP: LIPOPEPTIDE	surfactin	60	BGC0000433/86%
1.2	10,387	RiPP-LIKE	unknown	16	
1.3	41,208	OTHER	bacilysin	53	BGC0001184/100%
1.4	21,615	Sactipeptide, RiPP: Thiopeptide	subtilosin	29	BGC0000602/87%
1.5	44,322	NRPS	bacillibactin	43	BGC0000309/100%
		NRPS	paenibactin		BGC0000401/100%
1.6	39,542	T3PKS	unknown	60	
1.7	20,225	TERPENE	unknown	22	
1.8	73,995	NRPS, BETALACTONE	fengycin	62	BGC0001095/100%
		NRP	plipastatin		BGC0000407/100%
		NRP + polyketide	mycosubtilin		BGC0001103/90%
1.9	87,945	POLYKETIDE + NRP	bacillaene	57	BGC0001089/92%
		Polyketide: Trans-AT type I	macrobrevin		BGC0001470/93%
1.10	20,807	TERPENE	unknown	25	

**Table 3:** BGC comparison between AQ11M9 and closely related strains *B. halotolerans* strain V48-19 and PAMC26543.

Metabolite Type	Most Similar Known Cluster	AQ11M9	PAMC26543	*Halotolerans* v48-19
NRPS: Lipopeptide	surfactin	86%	86%	
RiPP-LIKE	unknown	+	−	−
OTHER	bacilysin	100%	100%	100%
SACTIPEPTIDE	Subtilosin A	87%	100%	100%
NRPS	bacillibactin	100%	100%	
NRPS	paenibactin	100%	100%	
T3PKS	unknown	+	−	−
TERPENE	unknown	+	−	−
NRPS, BETALACTONE	fengycin	100%	100%	
NRP	plipastatin	100%	100%	
Polyketide + NRP	bacillaene	92%	100%	
Polyketide: Trans-AT type I	macrobrevin	93%	53%	
TERPENE	unknown	+	−	−
bacteriocin	unknown	+	−	−

“+” observed in AQ11M9; “−” not present in closely related strains.

**Table 4:** Summary of biosynthetic gene cluster type NRP/Polyketide in AQ11M9 strain and its domain class.

BGC	NRP/Polyketide	Identity %	Align Length	E-Value	Domain Class
Surfactin	NRP	92	426	2.1 × 10^−231^	starter
		91	433	2.1 × 10^−231^	DCL
		89	445	2 × 10^−238^	epim
Bacilibactin	NRP	86	305	2.5 × 10^−152^	starter
		88	446	2 × 10^−229^	LCL
Fengycin	NRP	100	290	4.00 × 10^−169^	DCL
		98	436	4.50 × 10^−250^	starter
		98	429	8.20 × 10^−247^	LCL
		97	456	1.10 × 10^−264^	epim
Bacillaene	NRP/PK	88	418	1.20 × 10^−220^	Type I trans-AT

## Data Availability

The original contributions presented in the study are included in the article/Supplementary Material, further inquiries can be directed to the corresponding author/s.

## Supplementary Materials

The following supporting information can be downloaded at https://www.mdpi.com/article/10.3390/ijms251910408/s1.
