# Supplementary material for: Genome-Guided Identification of Surfactin-Producing Bacillus halotolerans AQ11M9 with Anti-Candida auris Potential
Source: Int J Mol Sci. 2024 Sep 27;25(19):10408. doi: 10.3390/ijms251910408 (PMC11476397; doi:10.3390/ijms251910408)
Supplement: Supplementary file 1 [file ijms-25-10408-s001.zip › ijms-3164853-supplementary.pdf]

Supplementary figures and tables.

Bacillus AQ11M9.

DOV

| Genome Name | Genome Status | Contigs | Isolation Country | Host Name | Collection Year | Completion Date | Distance | P value | K-mer Counts |
|---|---|---|---|---|---|---|---|---|---|
| Bacillus sp. PAMC26543 | Complete | 1 | Antarctica | | 2019 | 8/19/20 | 0.00920555 | 0 | 701/1000 |
| Bacillus halotolerans strain V48-19 | | 31 | USA | | 1974 | 1/4/18 | 0.00964976 | 0 | 690/1000 |
| Bacillus halotolerans strain N76 | WGS | 35 | China | Macaca fascicularis | 2019 | 6/16/21 | 0.00981378 | 0 | 686/1000 |
| Bacillus halotolerans strain P1 | Complete | 1 | USA | Pisolithus arhizus | 2014 | 12/16/20 | 0.0101459 | 0 | 678/1000 |
| Bacillus halotolerans strain MS50-18A | WGS | 61 | Mexico | | 2013 | 2/2/18 | 0.0101878 | 0 | 677/1000 |
| Bacillus halotolerans strain MBH1 | Complete | 1 | USA | | 2018 | 5/20/21 | 0.0102298 | 0 | 676/1000 |
| Bacillus halotolerans strain ZB201702 | Complete | 1 | China | | 2017 | 1/9/19 | 0.0110444 | 0 | 657/1000 |
| Bacillus halotolerans strain F41-3 | Complete | 1 | South Korea | | 2018 | 7/14/19 | 0.0110444 | 0 | 657/1000 |
| Bacillus mojavensis RRC 101 | WGS | 116 | | | | 6/3/13 | 0.0110882 | 0 | 656/1000 |
| Bacillus halotolerans strain LNXM37 | WGS | 34 | China | | 2005 | 3/14/18 | 0.0113974 | 0 | 649/1000 |
| Alkalihalobacillus halodurans strain ACCC 11011 | WGS | 23 | China | plant | 2021 | 7/13/21 | 0.0115313 | 0 | 646/1000 |
| Bacillus halotolerans strain XH-1 | Complete | 1 | China | | 2010 | 9/15/20 | 0.0117112 | 0 | 642/1000 |
| Bacillus sp. 7705b | WGS | 82 | | | | 9/11/17 | 0.0120757 | 0 | 634/1000 |
| Bacillus halotolerans strain LNXM78 | WGS | 25 | China | | 2005 | 3/14/18 | 0.0123068 | 0 | 629/1000 |
| Bacillus subtilis strain KKD1 | Complete | 1 | China | | 2010 | 6/16/20 | 0.0124 | 0 | 627/1000 |
| Bacillus halotolerans strain V44_23b | | 35 | USA | | 1974 | 1/4/18 | 0.0158335 | 0 | 559/1000 |
| Bacillus halotolerans strain RHF12 | WGS | 255 | Spain | | 2016 | 1/30/21 | 0.0170687 | 0 | 537/1000 |
| Bacillus halotolerans strain B19 | WGS | 139 | Algeria | | 2017 | 3/12/18 | 0.0174175 | 0 | 531/1000 |
| Bacillus halotolerans strain 86 | WGS | 52 | Tunisia | Limoniastrum monopetalum | 2017 | 3/12/18 | 0.0179508 | 0 | 522/1000 |
| Bacillus halotolerans strain 36 | WGS | 175 | Tunisia | Limoniastrum monopetalum | 2017 | 3/12/18 | 0.0181313 | 0 | 519/1000 |
| Bacillus halotolerans strain SpS5 | WGS | 54 | Tunisia | Solanum tuberosum | 2009 | 10/31/21 | 0.0187432 | 0 | 509/1000 |
| Bacillus halotolerans strain SpS5 | WGS | 54 | Tunisia | Solanum tuberosum | 2009 | 10/31/21 | 0.0187432 | 0 | 509/1000 |
| Bacillus halotolerans strain 72 | WGS | 697 | Tunisia | Limoniastrum monopetalum | 2017 | 3/12/18 | 0.0199514 | 0 | 490/1000 |

**Figure S1.** Whole-genome sequence analysis in PATRIC for taxonomy of AQ11M9.

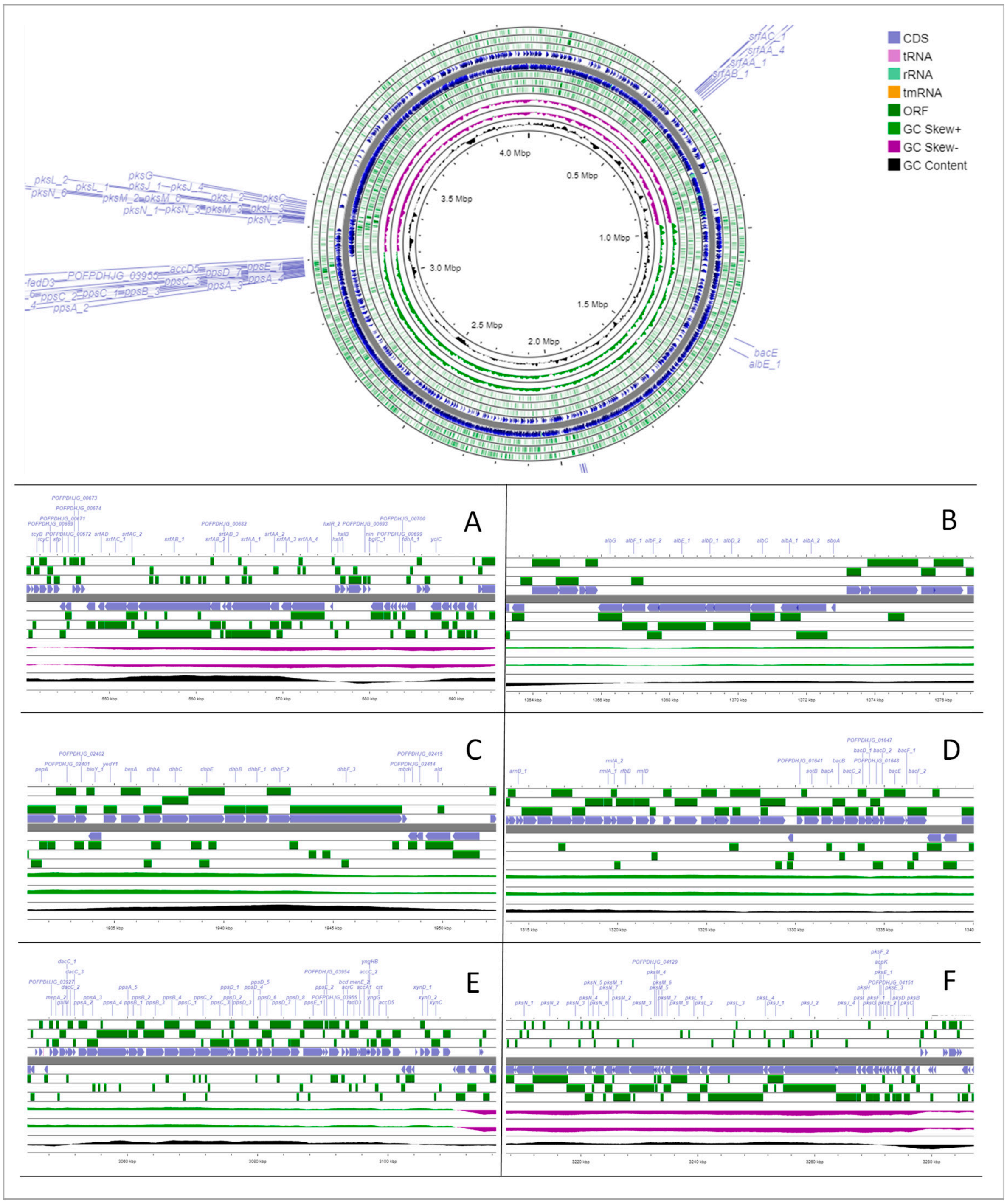

**Figure S2.** Top: Circular genome map for Bacillus halotolerans AQ11M9 strain represents all names of gene cluster. (A) Surfactin gene cluster; (B) subtilosin A gene cluster; (C) bacillibactin and paenibactin gene clusters; (D) bacilysin gene cluster; (E) fengycin, plipastatin and mycosubtilin gene clusters; (F) bacillaene and macrobrevin gene clusters.

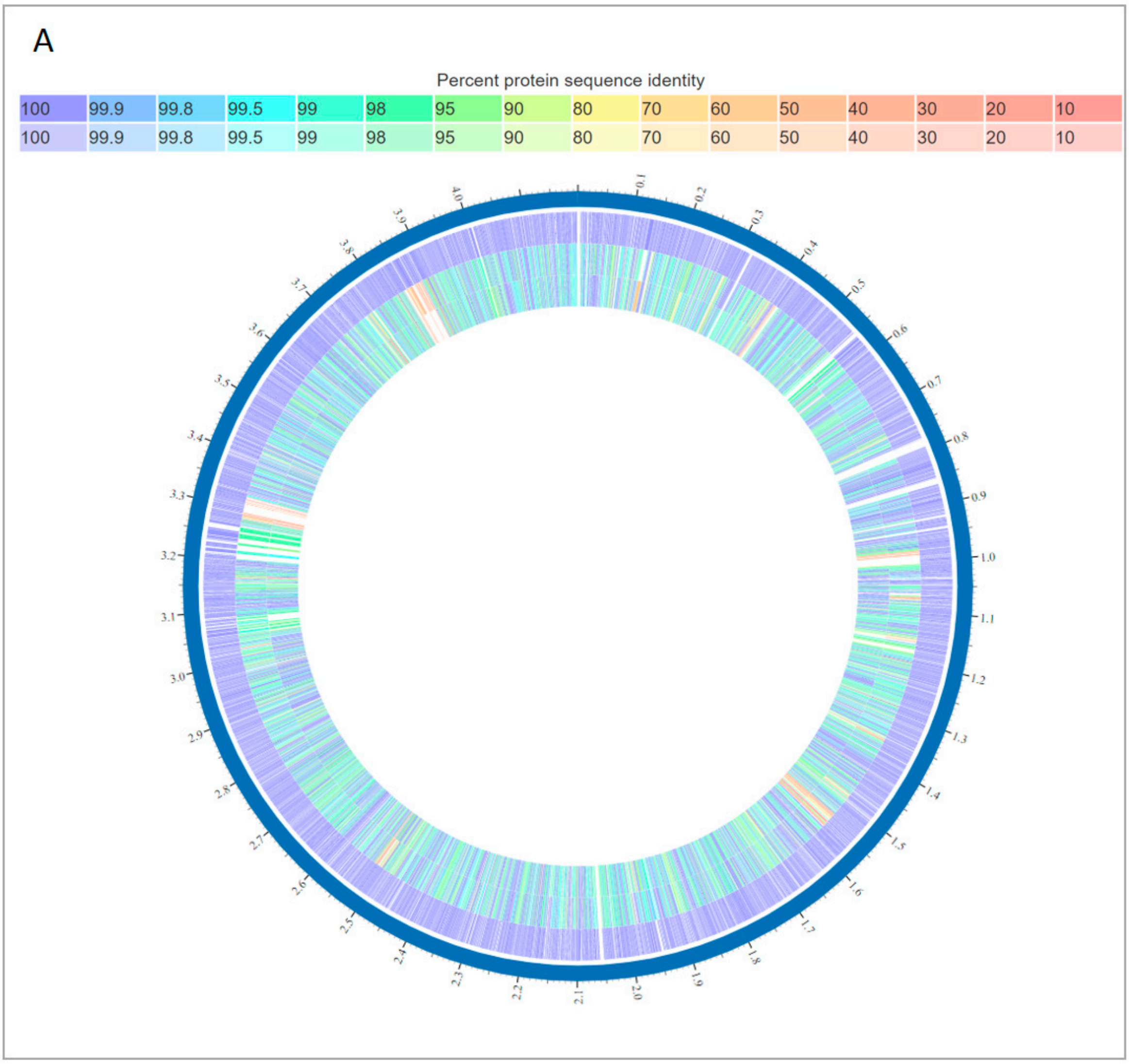


**Figure S3.** Proteome comparison from outside to inside AQ11M9, *B. halotolerans* strain V48-19 and PAMC26543.

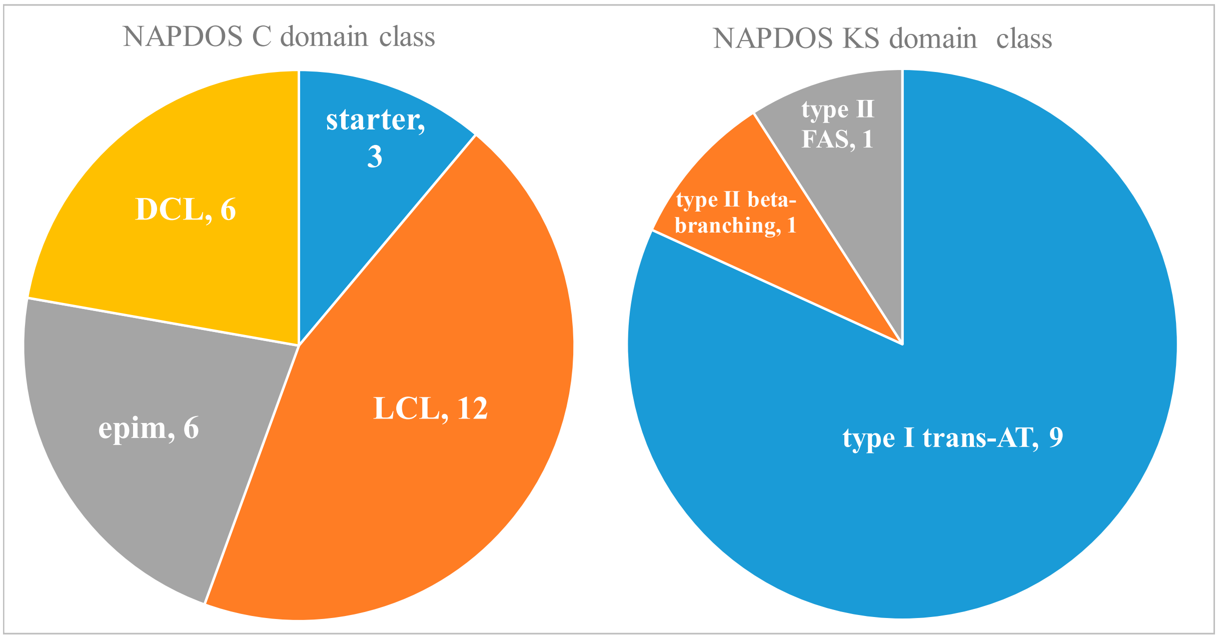


**Figure S4.** (A) NAPDOS organization of NRPS C domain in *B. halotolerans* AQ11M9 genome. Starter: first module of NRPS; Epim: epimerization domains which change the chirality from L- to D-amino acid in the last amino acid in the chain; LCL: catalyze establishment of a peptide bond between two L-amino acids; DCL: growing peptide linked with an L-amino acid ending with a D-amino acid. (B) NAPDOS arrangement of NRPS KS domain in *B. halotolerans* AQ11M9 genome. Type I trans AT is one of the Type I PKS classes; the acyltransferase (AT) activity is contributed via one or several independent proteins. Type II beta-branching is one of the Type II PKS classes responsible for the introduction of B-keto branches. Type II FAS, discrete, monofunctional proteins.

**Table S1.** Gene Family Statistics used for constructing whole genome phylogenetic tree of AQ11M9.

| PGFam | Align. Score | Align. Length | Num Seqs | Mean Sqr Freq | Prop Gaps | Used In Analysis | Product |
|---|---|---|---|---|---|---|---|
| PGF_00060409 | 21.07 | 723 | 66 | 0.784 | 0.043 | True | Translation elongation factor G |
| PGF_08675943 | 20.48 | 1281 | 66 | 0.572 | 0.088 | True | Transcription-repair coupling factor |
| PGF_03272313 | 18.92 | 1040 | 66 | 0.587 | 0.199 | True | DNA gyrase subunit A (EC 5.99.1.3) |
| PGF_00007041 | 18.17 | 663 | 66 | 0.706 | 0.076 | True | GTP-binding protein TypA/BipA |
| PGF_05195027 | 17.52 | 498 | 66 | 0.785 | 0.054 | True | ATP synthase beta chain (EC 3.6.3.14) |
| PGF_04569524 | 17.18 | 558 | 66 | 0.727 | 0.100 | True | ATP synthase alpha chain (EC 3.6.3.14) |
| PGF_00016338 | 15.76 | 486 | 66 | 0.715 | 0.132 | True | ATP-dependent Clp protease ATP-binding subunit ClpX |
| PGF_00052238 | 15.20 | 528 | 66 | 0.661 | 0.150 | True | Signal recognition particle protein Ffh |
| PGF_04333086 | 15.10 | 859 | 66 | 0.515 | 0.218 | True | DNA ligase (NAD(+)) (EC 6.5.1.2) |
| PGF_02226715 | 14.81 | 826 | 66 | 0.515 | 0.175 | True | ATP-dependent DNA helicase RecG (EC 3.6.4.12) |
| PGF_10300474 | 13.92 | 1267 | 66 | 0.391 | 0.444 | True | DNA topoisomerase I (EC 5.99.1.2) |
| PGF_00016393 | 13.77 | 282 | 66 | 0.820 | 0.020 | True | LSU ribosomal protein L2p (L8e) |
| PGF_00422271 | 13.28 | 372 | 66 | 0.689 | 0.152 | True | DNA-directed RNA polymerase alpha subunit (EC 2.7.7.6) |
| PGF_00007024 | 13.19 | 576 | 66 | 0.550 | 0.237 | True | GTP-binding protein EngA |
| PGF_02019462 | 12.38 | 404 | 66 | 0.616 | 0.147 | True | Phenylalanyl-tRNA synthetase alpha chain (EC 6.1.1.20) |
| PGF_02452671 | 11.98 | 533 | 66 | 0.519 | 0.195 | True | Cell division trigger factor (EC 5.2.1.8) |
| PGF_03004613 | 11.79 | 542 | 66 | 0.506 | 0.213 | True | Histidyl-tRNA synthetase (EC 6.1.1.21) |
| PGF_07063065 | 11.54 | 556 | 66 | 0.490 | 0.315 | True | Transcription termination protein NusA |
| PGF_00016357 | 11.47 | 246 | 66 | 0.731 | 0.060 | True | LSU ribosomal protein L1p (L10Ae) |
| PGF_00016443 | 10.78 | 185 | 66 | 0.792 | 0.035 | True | LSU ribosomal protein L5p (L11e) |
| PGF_06162930 | 10.67 | 554 | 66 | 0.453 | 0.185 | True | UDP-N-acetylmuramoyl-L-alanine--D-glutamate ligase (EC 6.3.2.9) |
| PGF_00015259 | 10.26 | 329 | 66 | 0.566 | 0.129 | True | ATP synthase gamma chain (EC 3.6.3.14) |
| PGF_04512522 | 10.00 | 216 | 66 | 0.680 | 0.052 | True | LSU ribosomal protein L4p (L1e) |
| PGF_00016444 | 9.63 | 184 | 66 | 0.710 | 0.030 | True | LSU ribosomal protein L6p (L9e) |
| PGF_00016343 | 9.62 | 146 | 66 | 0.796 | 0.045 | True | LSU ribosomal protein L16p (L10e) |
| PGF_00423533 | 9.48 | 388 | 66 | 0.481 | 0.252 | True | 4-diphosphocytidyl-2-C-methyl-D-erythritol kinase (EC 2.7.1.148) |
| PGF_03215471 | 9.34 | 327 | 66 | 0.516 | 0.113 | True | 16S rRNA (cytidine(1402)-2'-O)-methyltransferase (EC 2.1.1.198) |
| PGF_04807486 | 9.29 | 377 | 66 | 0.479 | 0.160 | True | tRNA dimethylallyltransferase (EC 2.5.1.75) |
| PGF_00048926 | 9.28 | 188 | 66 | 0.676 | 0.016 | True | Ribosome recycling factor |
| PGF_00049906 | 9.19 | 134 | 66 | 0.794 | 0.015 | True | SSU ribosomal protein S8p (S15Ae) |
| PGF_00689961 | 8.99 | 243 | 66 | 0.577 | 0.159 | True | Guanylate kinase (EC 2.7.4.8) |
| PGF_06180597 | 8.89 | 155 | 66 | 0.714 | 0.075 | True | LSU ribosomal protein L13p (L13Ae) |
| PGF_08582746 | 8.89 | 342 | 66 | 0.481 | 0.269 | True | 23S rRNA (guanosine(2251)-2'-O)-methyltransferase (EC 2.1.1.185) |
| PGF_06626131 | 8.84 | 172 | 66 | 0.674 | 0.080 | True | 2-C-methyl-D-erythritol 2,4-cyclodiphosphate synthase (EC 4.6.1.12) |
| PGF_03790040 | 8.81 | 333 | 66 | 0.483 | 0.254 | True | Ribonuclease III (EC 3.1.26.3) |
| PGF_00016445 | 8.35 | 129 | 66 | 0.736 | 0.060 | True | LSU ribosomal protein L7p/L12p (P1/P2) |
| PGF_00413295 | 8.23 | 409 | 66 | 0.407 | 0.249 | True | tRNA pseudouridine(55) synthase (EC 5.4.99.25) |
| PGF_00016358 | 8.20 | 129 | 66 | 0.722 | 0.085 | True | LSU ribosomal protein L20p |
| PGF_00413554 | 7.99 | 198 | 66 | 0.568 | 0.210 | True | tmRNA-binding protein SmpB |

| | | | | | | | |
|---|---|---|---|---|---|---|---|
| PGF_01213071 | 7.93 | 223 | 66 | 0.531 | 0.253 | True | LSU ribosomal protein L10p (P0) |
| PGF_00060478 | 7.65 | 278 | 66 | 0.459 | 0.375 | True | Translation initiation factor 3 |
| PGF_08518355 | 7.65 | 330 | 66 | 0.421 | 0.268 | True | ATP synthase F0 sector subunit a (EC 3.6.3.14) |
| PGF_04788810 | 7.58 | 224 | 66 | 0.506 | 0.152 | True | Peptidyl-tRNA hydrolase (EC 3.1.1.29) |
| PGF_06111020 | 7.45 | 251 | 66 | 0.470 | 0.159 | True | Thymidylate kinase (EC 2.7.4.9) |
| PGF_03174068 | 7.27 | 326 | 66 | 0.403 | 0.448 | True | Transcription antitermination protein NusG |
| PGF_00016377 | 6.69 | 140 | 66 | 0.566 | 0.256 | True | LSU ribosomal protein L24p (L26e) |
| PGF_04978890 | 6.55 | 130 | 66 | 0.575 | 0.214 | True | LSU ribosomal protein L21p |
| PGF_00020361 | 6.32 | 212 | 66 | 0.434 | 0.258 | True | Metal-dependent hydrolase YbeY, involved in rRNA and/or ribosome maturation and assembly |
| PGF_00435430 | 5.75 | 164 | 66 | 0.449 | 0.278 | True | Ribosomal silencing factor RsfA |
| PGF_03295678 | 5.72 | 466 | 66 | 0.265 | 0.580 | True | CDP-diacylglycerol--glycerol-3-phosphate 3-phosphatidyltransferase (EC 2.7.8.5) |
| PGF_00049901 | 5.14 | 141 | 66 | 0.433 | 0.315 | True | SSU ribosomal protein S6p |
